# 

**Published:** 1861-08

**Authors:** 


### CONTENTS.---AUGUST, 1861.

### ORIGINAL CONTRIBUTIONS.

PAGE
ART. I. Lectures on Military Surgery. By E. ANDREWS, A. M., M. D., Professor of Surgery, etc. Lecture IV., .......... 385
ART. II. Prolapsus Uteri with Ulceration. A Paper by DANIEL D. WAITE, M. D., Chicago, Illinois, .......... 397
ART. III. Spina Bifida. By VINDEX, .......... 403
ART. IV. An Experiment with Kerosolene—A new Anæsthetic. By E. ANDREWS, M. D., Professor of Surgery, etc., .......... 406
ART. V. Fractures of the Spine. Two Cases, by Dr. G. K. AMERMAN, Chicago, Ill., .......... 409

### THE CLINIQUE.

MERCY HOSPITAL—Surgical Wards. Service, Prof. E. ANDREWS, A. M., M. D. Operation for Removal of Malignant Epulis, .......... 413
CHICAGO MEDICAL DISPENSARY.—Service, Prof. WM. H. BYFORD, M. D., Attending Physician. Ague Cake, .......... 415
Triple Hernia. Service, Prof. E. ANDREWS, Attending Surgeon, .......... 419

### MEDICAL SOCIETIES.

CHICAGO MEDICAL SOCIETY.—Report of July Meeting, .......... 420
Cases of Arsenical Poisoning. Reported by N. S. DAVIS, M. D., Prof. of Practical Medicine, etc., .......... 421
DEWITT COUNTY MEDICAL SOCIETY.—Report of July Meeting, .......... 424

### SELECTIONS.

Arseniate of Soda in Scrofula—BOUCHUT, .......... 425
Anæsthetics—Theory of their Action—JACKSON, .......... 428
FOREIGN ITEMS.—Action of Chloroform on the Blood—SANSOM, .......... 431
Consumption of Alcoholic Stimulants in Hospitals—GAIRDNER, .......... 432
Coffee as a Remedy for Whooping-Cough—GUYOT, .......... 433
Prolapsed Bowel Treated by Subcutaneous Strychnia Injections—DOLBEAN, .......... 434

### BOOK NOTICES.

A Treatise on the Practice of Medicine. By EDWIN R. MAXON, M. D., etc., .......... 434
OUR EXCHANGES.—BRAITHWAITE'S Retrospect, .......... 435
North-American Medico-Chirurgical Review ...London Lancet, .......... 436
American Journal of Medical Sciences, .......... 437

### EDITORIAL.

Clinical Instruction and Medical Schools, .......... 437
Cholera Infantum—Summer Complaints and Teething, .......... 438
COMMUNICATIONS.—Kerosolene—Letter from Dr. DICKINSON, .......... 439
The New Splints for Hip Disease, .......... 444
Surgeons for the Illinois Volunteers, .......... 446

